# Predicting Coronary Artery Aneurysms in Kawasaki Disease at a North American Center: An Assessment of Baseline *z* Scores

**DOI:** 10.1161/JAHA.116.005378

**Published:** 2017-05-31

**Authors:** Mary Beth F. Son, Kimberlee Gauvreau, Susan Kim, Alexander Tang, Fatma Dedeoglu, David R. Fulton, Mindy S. Lo, Annette L. Baker, Robert P. Sundel, Jane W. Newburger

**Affiliations:** ^1^ Division of Immunology Boston Children's Hospital Boston MA; ^2^ Department of Cardiology Boston Children's Hospital Boston MA; ^3^ Department of Pediatrics Harvard Medical School Boston MA; ^4^ University of California San Francisco School of Medicine San Francisco CA

**Keywords:** aneurysm, echocardiography, Kawasaki disease, outcome, Aneurysm, Clinical Studies

## Abstract

**Background:**

Accurate risk prediction of coronary artery aneurysms (CAAs) in North American children with Kawasaki disease remains a clinical challenge. We sought to determine the predictive utility of baseline coronary dimensions adjusted for body surface area (*z* scores) for future CAAs in Kawasaki disease and explored the extent to which addition of established Japanese risk scores to baseline coronary artery *z* scores improved discrimination for CAA development.

**Methods and Results:**

We explored the relationships of CAA with baseline *z* scores; with Kobayashi, Sano, Egami, and Harada risk scores; and with the combination of baseline *z* scores and risk scores. We defined CAA as a maximum *z* score (zMax) ≥2.5 of the left anterior descending or right coronary artery at 4 to 8 weeks of illness. Of 261 patients, 77 patients (29%) had a baseline zMax ≥2.0. CAAs occurred in 15 patients (6%). CAAs were strongly associated with baseline zMax ≥2.0 versus <2.0 (12 [16%] versus 3 [2%], respectively, *P*<0.001). Baseline zMax ≥2.0 had a C statistic of 0.77, good sensitivity (80%), and excellent negative predictive value (98%). None of the risk scores alone had adequate discrimination. When high‐risk status per the Japanese risk scores was added to models containing baseline zMax ≥2.0, none were significantly better than baseline zMax ≥2.0 alone.

**Conclusions:**

In a North American center, baseline zMax ≥2.0 in children with Kawasaki disease demonstrated high predictive utility for later development of CAA. Future studies should validate the utility of our findings.

**Clinical Perspective**

**What Is New?**

- Accurate prediction of CAAs in North American children with KD remains a clinical challenge.
- A baseline coronary artery *z* score ≥2.0 had higher predictive utility for aneurysm development than demographic or laboratory variables or established Japanese risk scores.

**What Are the Clinical Implications?**

- Because baseline *z* scores are quantitative measures that are obtained in the routine care of patients with KD, our findings raise the possibility that *z* scores may be used as an imaging biomarker for the identification of high‐risk patients with KD in North American populations.
- Early identification of high‐risk children may allow for tailoring of treatment and design of well‐powered clinical trials to assess efficacy of treatment regimens.
- Future studies should validate the utility of our findings.

## Introduction

Kawasaki disease (KD) is a vasculitis of medium and small muscular arteries that primarily affects young children and has a predilection for the coronary arteries. Although the majority of children with KD respond well to treatment with intravenous immunoglobulin (IVIG), some develop coronary artery aneurysms (CAAs), a potentially devastating consequence of the disease that is associated with significant morbidity.^1^, ^2^, ^3^ Prior studies have reported that higher coronary dimensions on the baseline echocardiogram (ie, at diagnosis) are more common among children in whom CAAs evolve.^4^, ^5^ Other risk factors for CAAs include the persistence or recrudescence of fever after a single dose of IVIG (referred to as *IVIG resistance*),^6^, ^7^ male sex,^8^, ^9^ age ≤12 months,^9^, ^10^ Asian race,^8^, ^11^ and delay in diagnosis.^12^, ^13^, ^14^

Three risk scores for the prediction of IVIG resistance have been developed in Japan: the Kobayashi,^15^ Egami,^16^ and Sano^17^ risk scores. The Harada risk score was devised to identify indications for treatment with IVIG.^18^ Demographic, clinical, and laboratory data points constitute the scores (Table 1). Sleeper et al assessed the performance of the Kobayashi, Sano, and Egami risk scores in the Pediatric Heart Network data set and found that they had low sensitivity and moderate specificity for predicting IVIG resistance in a North American cohort.^19^ Tremoulet et al described similar findings with the application of the Egami risk score to a cohort from San Diego, California.^20^ The Harada risk score has been applied to a US population and was found to be 90% sensitive for identifying children at high risk of CAAs but had low specificity (51%) and low positive predictive value (PPV; 19%).^21^

**Table 1:** Characteristics of Japanese Risk Scores

Kobayashi, Cutoff for High Risk: ≥4	Sano, Cutoff for High Risk: ≥2	Egami, Cutoff for High Risk: ≥3	Harada, Cutoff for High Risk: ≥4
Fever ≤4 d (2)^a^	…	Fever ≤4 d (1)	…
Age ≤12 mo (1)	…	Age ≤6 mo (1)	Age ≤12 mo (1)
…	…	…	Male sex (1)
C‐reactive protein ≥10 mg/dL (1)	C‐reactive protein ≥7 mg/dL (1)	C‐reactive protein ≥8 mg/dL (1)	C‐reactive protein >3 mg/dL (1)
Platelets ≤300 000/mm^3^ (1)	…	Platelets ≤300 000/mm^3^ (1)	Platelets <350 000/μL (1)
AST ≥100 IU/L (2)	AST ≥200 IU/L (1)	ALT ≥80 IU/L (2)	…
Sodium ≤133 mmol/L (2)	…	…	…
Neutrophils ≥80% (2)	…	…	White blood cell >12 000/μL (1)
…	Total bilirubin ≥0.9 mg/dL (1)	…	…
…	…	…	Albumin <3.5 g/L (1)
			Hematocrit <35% (1)

ALT indicates alanine aminotransferase; AST, aspartate aminotransferase.

a Number in parentheses indicates number of points awarded for each component to calculate risk score.

Because the performance of the established risk scores in North American cohorts has been unsatisfactory, better methods of identifying children at the highest risk of aneurysm formation are needed. Such identification would benefit the individual patient via early risk identification and tailoring of treatment and potentially allow for the design of well‐powered treatment trials to determine optimal therapy in KD.

In the current single‐center study, we sought to determine the predictive utility of baseline echocardiography for later CAA and to assess test characteristics of baseline coronary dimensions adjusted for body surface area (ie, *z* scores). We further explored the extent to which addition of variables in established Japanese risk scores to baseline coronary artery *z* scores could improve discrimination between children who develop coronary aneurysms and those who do not.

## Methods

### Identification of Cohort

Demographic, clinical, laboratory, and echocardiographic data were abstracted for patients diagnosed with KD at a single academic center from January 1, 2006, through May 1, 2014. Race and ethnicity were extracted from the electronic medical record, which is populated with information provided by the parents on registration with the hospital. We collected this information because prior studies have indicated that patients of Asian descent^8^, ^11^ or Hispanic ethnicity^11^ are at increased risk of poor coronary outcomes. Patients with incomplete KD^22^ were included. We excluded patients who had at least one of the following criteria: (1) a second episode of KD; (2) presentation at our center for a second opinion; (3) first evaluation at our center in the subacute phase of illness; (4) no or unknown treatment with IVIG; (5) missing all laboratory data for calculation of established Japanese risk scores; (6) first laboratory studies obtained ≥10 days after fever onset or ≥1 day after IVIG administration; (7) no echocardiography at baseline, meaning obtained either before IVIG administration or within 2 days of first IVIG treatment; or (8) no echocardiographic data available in the 4 to 8 weeks after illness onset.

Coronary artery dimensions from the left anterior descending artery and the proximal right coronary artery were normalized for body surface area (*z* scores) using the Boston formula.^5^ Coronary artery *z* scores were calculated for baseline studies and for studies obtained 4 to 8 weeks after illness onset. The maximum *z* score (zMax) was defined as the larger *z* score of the left anterior descending or right coronary artery on a particular echocardiogram. CAAs were defined as a zMax ≥2.5 of the proximal right coronary artery and/or proximal left anterior descending artery at 4 to 8 weeks following fever onset. If multiple echocardiograms were obtained in the 4‐ to 8‐week time period, we utilized the largest zMax from this window. For those patients with CAA at 4 to 8 weeks, subsequent echocardiograms were assessed for persistence or regression of the CAA.

### Risk Scores

Clinical, demographic, and laboratory data points from our cohort were used for calculation of published Japanese risk scores^15^, ^16^, ^17^, ^18^ (Table 1). Cut points specified by the authors of each risk score were applied to determine whether a patient's risk was considered to be high or low.

### Analysis

Demographic characteristics and echocardiographic data were summarized using frequencies and percentages for categorical variables and medians with ranges for continuous variables. We compared proportions of patients with subsequent CAA for those with baseline zMax ≥2.0 versus <2.0, baseline zMax of ≥2.5 versus <2.5, and baseline zMax of ≥3.0 versus <3.0 using the Fisher exact test. Similar analyses were performed for each Japanese risk score, comparing high versus low risk groups. Sensitivity, specificity, PPV, and negative predictive value for CAA were calculated for baseline zMax ≥2.0 and for each risk score. A *z* score >2.0 was not selected because we wanted to minimize the false‐negative rate of our cutoff and maximize the detection of children who are at risk for progressive coronary artery dilation. Odds ratios for CAA were estimated using logistic regression models; the discrimination for each model was quantified using the area under the receiver operating characteristic curve (C statistic). The C statistic is a measure of goodness‐of‐fit for binary outcomes in a logistic regression model. A C statistic of 0.5 means that the model has no ability to discriminate between patients who do and do not experience the outcome; a value of 1 means that the model predicts the outcome perfectly. Increases in the C statistic when each Japanese risk score was added to a model that already contained baseline zMax ≥2.0 were calculated. The improvement in risk prediction when each risk score was included in combination with baseline zMax was also assessed using the category‐free net reclassification improvement (NRI).^23^ The NRI is the sum of the net percentage of patients with CAA for whom the predicted probability of the outcome increases when risk score is added to baseline zMax and the net percentage of patients without CAA for whom the predicted probability of the outcome decreases when risk score is added; these net percentages are also reported. The NRI takes values from −2 to 2, with higher positive values representing improved risk prediction. Additional demographic and laboratory characteristics were compared for patients with and without CAA, using the Wilcoxon rank sum test for continuous variables and the Fisher exact test for categorical variables. Associations of these variables with CAA after adjusting for baseline zMax were assessed using multivariable logistic regression models. Forward stepwise selection was used, and *P*<0.05 was required for retention in the final model.

Approval from our institutional review board with a waiver of informed consent was obtained for this project.

## Results

A total of 504 patients were diagnosed with KD at our center during the study period. After the exclusion criteria were applied, 261 patients remained for analysis and formed the cohort for this study (Table 2). All patients were treated within 10 days of fever onset; 67 patients (26%) required retreatment for IVIG resistance (persistent or recrudescent fever 36 hours after completion of first IVIG) and received >1 dose of IVIG (Table 2). Of those patients retreated with IVIG, 18 patients were also treated with corticosteroids and 5 patients with infliximab for persistent fever and/or expanding coronary arteries. A comparison of baseline characteristics between the cohort and the 70 patients who were excluded due to lack of echocardiographic data at 4 to 8 weeks after illness onset revealed no significant differences (Table S1).

**Table 2:** Baseline Characteristics of Cohort (n=261)

Variable	n or median, (% or IQR)
Age at fever onset, y	3.2 (1.7, 5.2)
Male sex	169 (65)
Race	
White	165 (63)
Black	29 (11)
Asian	44 (17)
Other, including >1 race	19 (7)
Not reported	4 (2)
Hispanic	35 (13)
Congenital heart disease	6 (2)
Days of fever	7 (6, 8)
Clinical criteria for KD	
≤3	70 (27)
4	127 (49)
5	64 (25)
Retreatment with IVIG	67 (26)
Days from first to second treatment	2 (2, 3)

IQR indicates interquartile range; IVIG, intravenous immunoglobulin; KD, Kawasaki disease.

Baseline echocardiography was obtained either before IVIG administration or within 2 days of first IVIG treatment. Nearly 30% of patients (n=77) had a zMax ≥2.0, 21% had a *z* score ≥2.5 (n=55), and 15% (n=38) had a *z* score ≥3.0 (Figure). By 4 to 8 weeks after fever onset, 15 patients (6%) had CAA. Of those, 12 had a zMax ≥2.0 at baseline. For nearly half of the patients, (114/261, 44%), the baseline zMax was the largest *z* score recorded for that patient within 8 weeks of illness onset. Seven of the 15 patients with aneurysms at 4 to 8 weeks of illness had aneurysms that persisted at latest follow‐up (mean follow up: 67±32 months; range: 19–98 months).

**Figure 1:**
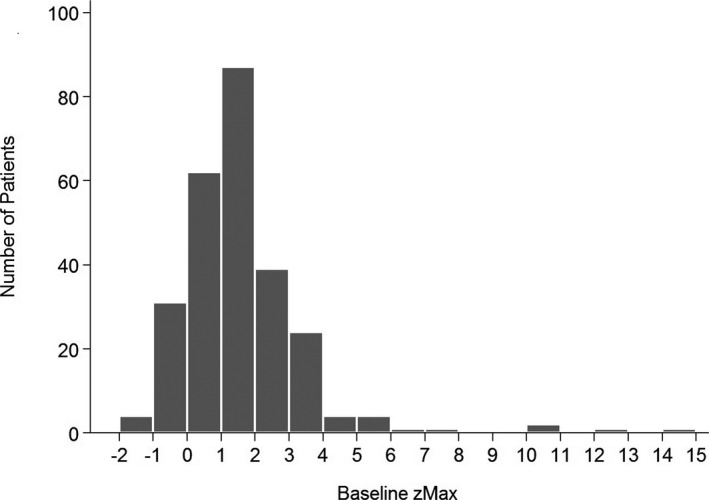
Histogram of baseline maximum *z* score (zMax) for our cohort (n=261).

Baseline zMax ≥2.0 was strongly associated with aneurysm development compared with a baseline zMax <2.0 (16% versus 2%, *P*<0.001; Table 3), with a C statistic of 0.77 (95% confidence interval, 0.66–0.88; Table 4). We also calculated sensitivity, specificity, PPV, and negative predictive value for baseline zMax ≥2.0 and for the high‐risk category of each of the risk scores (Table 5). Sensitivity was quite good for a baseline zMax ≥2.0 (80%) and excellent for the Harada risk score (100%) but poor for the Kobayashi, Sano, and Egami risk scores (21%, 13%, and 21%, respectively). A baseline zMax ≥2.0 had a specificity of 74%. Specificity ranged from low to moderate across the Japanese risk scores, with the Harada risk score having the lowest specificity (36%). PPV was low for a baseline zMax ≥2.0 and for all risk scores, and conversely, negative predictive value was >90% for a baseline zMax ≥2.0 and for all risk scores.

**Table 3:** Baseline zMax and Risk Scores as Predictors of CAAs at 4 to 8 Weeks

	n^a^	Patients With CAA, n (%)	*P* Value
Baseline zMax^b^			
<2.0	184	3 (2)	<0.001
≥2.0	77	12 (16)	
Kobayashi			
Low risk (0–3)	163	11 (7)	0.39
High risk (≥4)	83	3 (4)	
Sano			
Low risk (0–1)	203	11 (5)	0.48
High risk (≥2)	39	3 (8)	
Egami			
Low risk (0–2)	201	13 (7)	0.74
High risk (≥3)	54	2 (4)	
Harada			
Low risk (0–3)	88	0 (0)	0.003
High risk (≥4)	170	15 (9)	

CAA indicates coronary artery aneurysm; zMax, maximum *z* score.

a Varying numbers of patients were analyzed for risk scores because not all patients had complete laboratory data for all scores.

b zMax is the larger of baseline *z* scores of the left anterior descending artery and right coronary artery.

**Table 4:** C Statistics, Incremental AUC, and NRI of Baseline *z* Scores and Japanese Risk Scores

					Net Percentage of Patients	
	C Statistic	Adding High Risk to Baseline zMax^a^ ≥2.0 (95% CI)	Increase in AUC (*P* Value)	Category‐Free NRI	With CAA, Assigned Higher Predicted Risk Per Risk Score, %	Without CAA, Assigned Lower Predicted Risk Per Risk Score, %
Baseline zMax ≥2.0	0.77	···	···	···	···	···
Kobayashi high‐risk score	0.57	0.83 (0.74–0.91)	0.03 (*P*=0.14)	0.26	57.1	−31.0
Sano high‐risk score	0.53	0.77 (0.63–0.90)	0.02 (*P*=0.26)	0.17	73.3	−56.7
Egami high‐risk score	0.54	0.79 (0.69–0.89)	0.01 (*P*=0.85)	−0.11	57.1	−68.4
Harada high‐risk score	Cannot estimate c statistic—no CAA in low risk group			0.72	100	−27.6

AUC indicates area under the curve; CAA, coronary artery aneurysm; CI, confidence interval; NRI, category‐free net reclassification improvement; zMax, maximum *z* score.

a zMax is the larger of the baseline *z* scores of the left anterior descending artery and right coronary artery.

**Table 5:** Test Characteristics of Baseline *z* Scores and Risk Scores

	Sensitivity (15 With CAA)	Specificity (246 Without CAA)	Positive Predictive Value	Negative Predictive Value
Baseline zMax ≥2.0	80%	74%	16%	98%
Kobayashi high risk	21%	66%	4%	93%
Sano high risk	21%	84%	8%	95%
Egami high risk	13%	78%	4%	94%
Harada high risk	100%	36%	9%	100%

CAA, coronary artery aneurysm; zMax, maximum *z* score.

In contrast to baseline zMax ≥2.0, stratification of patients in high‐ versus low‐risk groups using the criteria of the Kobayashi, Sano, and Egami risk scores revealed no association between high‐risk status and CAA (Table 3), and they demonstrated poor discrimination for CAA development because they each had a C statistic <0.6 (Table 4). The Harada score, however, was significantly associated with CAA; all aneurysms occurred in the high‐risk group, with none in the low‐risk group (9% versus 0%, *P*=0.003; Table 3). A high‐ versus low‐risk status per the Harada score was not predictive of aneurysm development in children who had a baseline zMax <2 (2.6% versus 0%, *P*=0.3). In contrast, a high‐ versus low‐risk Harada score in children with a baseline zMax ≥2 was significantly associated with CAA (21.5% versus 0%, *P*=0.03).

To determine whether addition of the Japanese risk scores to baseline zMax improved CAA prediction, we constructed bivariate models that each included baseline zMax ≥2.0 plus high‐risk status for one of the risk scores. The absence of aneurysm patients in the low‐risk group for the Harada risk score precluded calculation of a C statistic, but NRI could still be estimated. C statistics for the bivariate models including Kobayashi, Sano, and Egami risk scores were 0.83 (95% confidence interval, 0.74–0.91), 0.77 (95% confidence interval, 0.63–0.90) and 0.79 (95% confidence interval, 0.69–0.89), respectively (Table 4); increases in areas under the curve were not statistically significant. NRI when the Kobayashi, Sano, and Egami risk scores were added to baseline zMax was low, indicating that addition of these risk scores did not improve our ability to predict CAA beyond zMax alone.

Laboratory components of the risk scores and demographic characteristics were analyzed for association with CAA. Patients with CAA at 4 to 8 weeks after fever onset, compared with those without CAA, had higher white blood cell counts and C‐reactive protein as well as lower albumin (Table S2). Patients of young age at fever onset (≤6 months and <1 year) compared with older children were significantly more likely to develop CAA, and Asian children compared with other races were also significantly more likely to develop CAA (Table S2).

In multivariable logistic regression, inclusion of Asian race with baseline echo zMax ≥2.0 increased the C statistic from 0.77 to 0.81; inclusion of age at fever onset ≤6 months further increased discrimination to 0.85. Calibration of this model was satisfactory (Hosmer‐Lemeshow, *P*=0.89). Of these variables, baseline zMax ≥2 had the highest discrimination for predicting CAA development.

## Discussion

KD is the leading cause of acquired heart disease in the developed world,^22^ and the development of persistent CAA in childhood is associated with high morbidity through adulthood.^2^, ^24^, ^25^ Over time, CAA can remodel to normal lumen diameter (so‐called regression) or to smaller dimensions via myofibroblastic proliferation,^26^ remain stable, develop stenoses, or develop thromboses. Coronary aneurysms, particularly those that are not giant, may remodel to normal lumen diameter, but this so‐called regression of aneurysms may be accompanied by abnormalities of vessel wall reactivity and intimal thickness^27^, ^28^, ^29^, ^30^ as well as cardiovascular events later in life.^31^, ^32^ To institute effective therapies early enough to prevent coronary arterial wall damage and its attendant morbidity, high‐risk children should ideally be identified at the time of diagnosis of KD. In the current study, we explored predictors of CAA at 4 to 8 weeks of illness, when dimensions of most aneurysms stabilize or begin to diminish in size and long‐term management guidelines replace acute management strategies.^22^ Moreover, children with normal echocardiographic findings at 4 to 8 weeks rarely have abnormalities 1 year after KD.^33^, ^34^ We found that zMax ≥2.0 on baseline echocardiography is highly associated with the presence of CAA at 4 to 8 weeks of illness, offering a possible imaging biomarker to improve the outcomes of children with KD.

Our findings build on earlier reports suggesting that patients who developed coronary aneurysms had higher baseline zMax scores. Using data from the Pediatric Heart Network's trial of pulsed‐dose corticosteroid therapy, McCrindle et al noted that a baseline zMax ≥2.5 predicted subsequent *z* scores ≥2.5 over 5 weeks of follow‐up in more than three quarters of patients.^5^ Dominguez et al reported that 4 in 5 children who developed coronary aneurysms had coronary artery abnormalities on initial echocardiogram; coronary abnormalities were first detected at a median of illness day 7 (interquartile range: 5–8 days).^4^

We also explored whether Japanese risk scores for IVIG resistance could predict the occurrence of CAA. The Kobayashi, Sano, and Egami risk scores were each developed in Japan to identify children at high risk for nonresponse to IVIG treatment, which in turn is highly associated with the development of CAA.^35^, ^36^, ^37^, ^38^ These IVIG resistance risk scores did not accurately predict which children in our cohort developed CAA, nor did they improve discrimination beyond that of a baseline zMax ≥2.0 alone in our study population. Consistent with our data, prior studies have indicated that these established Japanese scores for IVIG resistance do not perform well in North American mixed‐ethnicity cohorts.^19^, ^20^

The Harada score was designed to predict CAA rather than IVIG resistance. Among children with baseline zMax ≥2.0, the Harada score placed all patients with CAA into the high‐risk group. Historically, the Harada score was constructed to identify patients who warranted treatment with IVIG at a time when IVIG treatment was not the standard of care in Japan.^18^ As such, the cutoff point for high versus low risk was set for maximum sensitivity, which was recapitulated in our population, as we found 100% sensitivity for the Harada score. Conversely, it had very low specificity. Because there were no CAA patients in the low‐risk group, the Harada score could not be tested in a logistic regression model and discrimination could not be assessed; however, it improved risk prediction in an NRI analysis. Application of the Harada score in a larger population may allow for further testing of its performance.

Baseline zMax ≥2.0 had good sensitivity at 80% and reasonable specificity at 74%. The Kobayashi, Sano, and Egami scores had very low sensitivity, which limits their clinical applicability, given the need to identify at‐risk patients. PPV was low for baseline zMax ≥2.0 and for the risk scores due to the low prevalence of the outcome of CAA as well as the expected finding of regression from a high baseline *z* score to a normal *z* score in some patients. Negative predictive value was >90% for all variables, as baseline *z* score <2.0 and low risk status per the Japanese risk scores were associated with low probability of developing CAA at 4 to 8 weeks after fever onset.

Using multivariable analysis, we assessed the association of clinical and laboratory variables in the Japanese risk scores as well as baseline zMax ≥2.0 with CAA development in our population. We found that a model consisting of baseline zMax ≥2.0, age at time of illness onset ≤6 months, and Asian race provided excellent discrimination, with a C statistic of 0.85. This finding may provide a clinically useful tool but requires validation in an independent data set. Furthermore, the modeling may be limited by the relative rarity of Asian race in the United States and KD patients aged ≤6 months.

Our analysis had certain limitations. Our study was a retrospective analysis, which led to variation in timing of follow‐up echocardiograms. Furthermore, the low number of patients with CAA limited our ability to perform multivariable analyses or to reliably test the discrimination of larger *z* scores. We had limited power to determine whether earlier diagnosis (ie, on days 3–5) affected the strength of the predictive value of baseline coronary *z* scores. Missing laboratory data diminished the total number of participants available; therefore, the power of this study to identify children at increased risk of developing CAA was limited. Similarly, we excluded 70 patients from the study because they did not have outcome data from echocardiography at 4 to 8 weeks after illness onset. Because patients with CAA are more likely to be followed closely, our studied population might have been skewed toward a more severe phenotype. Nevertheless, baseline characteristics of the excluded patients, including baseline *z* scores, were not significantly different from those of the included patients, making spectrum bias somewhat less likely. Baseline coronary artery *z* scores might have been associated with later measurements because longitudinal data tend to be correlated; however, coronary arterial wall enlargement can progress rapidly in some patients with KD within the first 4 to 8 weeks. Patients with expanding coronary artery dimensions generally received adjunctive therapies, which could have biased our study toward less positive results by improving 4‐ to 8‐week coronary artery outcomes, but none of these additional therapies have been shown to improve coronary artery outcomes.^39^, ^40^ The generalizability of our results may be somewhat limited in centers that are unable to obtain echocardiography either prior to or within 48 hours of receiving IVIG. Future work could include evaluation of echocardiographic data beyond this time period. Finally, validation of our findings is important to avoid unnecessary escalation of primary treatment in children with KD.

In summary, a baseline zMax ≥2.0 had higher predictive utility for CAA development than any other variable tested, including established Japanese risk scores and demographic and laboratory variables. Moreover, the addition of Japanese risk scores to baseline zMax did not appreciably improve discrimination. Because baseline *z* scores are quantitative measures that are obtained in the routine care of patients with KD, our findings raise the possibility that *z* scores may be used as an imaging biomarker for the identification of high‐risk KD patients in North American populations, both for tailoring treatment of individual patients and potentially for enrollment in therapeutic trials.

## Sources of Funding

This work was supported by the McCance Family Foundation, whose members had no role in the design and conduct of the study; collection, management, analysis, and interpretation of the data; preparation, review, or approval of the manuscript; and decision to submit the manuscript for publication.

## Disclosures

None.

## Supporting information


**Table S1.** Comparison of Patients Included Versus Excluded for Lack of Echocardiography at 4 to 8 Weeks After Illness Onset
**Table S2.** Demographic and Laboratory Variables Associated With Coronary Artery Aneurysm
