# Supplementary material for: Predicting Coronary Artery Aneurysms in Kawasaki Disease at a North American Center: An Assessment of Baseline z Scores
Source: J Am Heart Assoc. 2017 May 31;6(6):e005378. doi: 10.1161/JAHA.116.005378 (PMC5669166; doi:10.1161/JAHA.116.005378)
Supplement: Supplementary file 1 — Table S1. Comparison of Patients Included Versus Excluded for Lack of Echocardiography at 4 to 8 Weeks After Illness Onset Table S2. Demographic and Laboratory Variables Associated With Coronary Artery Aneurysm [file JAH3-6-e005378-s001.pdf]

SUPPLEMENTAL MATERIAL

Table S1. Comparison of patients included *vs.* excluded due to lack of echocardiography at 4-8 weeks after illness onset

	**Excluded (n=70)**	**Included (n=261)**	**p value**
Age at Fever Onset (years)	3.6 (1.8, 6.1)	3.2 (1.7, 5.2)	0.28
Age at Fever Onset: <1 year	7 (10%)	35 (13%)	0.73
Male Sex	41 (59%)	169 (65%)	0.40
Hispanic Ethnicity	16 (23%)	35 (13%)	0.13
Asian Race	12 (17%)	50 (19%)	0.86
Days of Fever	7 (6, 8)	7 (6, 8)	0.73
IVIG Retreatment	14 (20%)	67 (26%)	0.35
Baseline Echo Max z score:			
• <2.5	60 (86%)	206 (79%)	0.52
• 2.5 to 2.99	3 (4%)	17 (7%)	
• >3.0	7 (10%)	38 (15%)	

Table S2. Demographic and laboratory variables associated with CAA

at 4-8 weeks after illness onset

**Characteristic**	**CAA (n=15) vs. no CAA (n=246)**	**p value**
White Blood Cell Count (median)	20 WBC/mL *vs*. 14 WBC/mL,	p=0.002
C-Reactive Protein (median)	14 mg/dL *vs*. 8 mg/dL	P=0.01
Albumin (median)	2.9 g/dL *vs*. 3.4 g/dL	p<0.001
Age < 1 year	33% (n=5) *vs*. 12% (n=30)	p=0.04
Age ≤ 6 months	20% (n=3) *vs* 4% (n=9)	p=0.02
Asian race	47%(n=7) *vs*. 17% (n=43)	p=0.05
